# Phospholipase D1 protein coordinates dynamic assembly of HIF-1α-PHD-VHL to regulate HIF-1α stability

**DOI:** 10.18632/oncotarget.2613

**Published:** 2014-10-21

**Authors:** Mi Hee Park, Kang-Yell Choi, Yunjin Jung, Do Sik Min

**Affiliations:** ^1^ Department of Molecular Biology, Pusan National University, Busan, Republic of Korea; ^2^ Department of Biotechnology, College of Life Science and Biotechnology, Yonsei University, Seoul, Republic of Korea; ^3^ Translational Research Center for Protein Function Control, Yonsei University, Seoul, Republic of Korea; ^4^ College of Pharmacy, Pusan National University, Busan, Republic of Korea

**Keywords:** Phospholipase D1, Hypoxia-inducible factor-1α, Prolyl hydroxylase, Von Hippel-Lindau, Pleckstrin homology domain

## Abstract

Hypoxia-inducible factor-1α (HIF-1α) is a master transcriptional regulator of cellular response to hypoxia. In normoxia, HIF-1α is degraded through the prolyl hydroxylase (PHD) and von Hippel-Lindau (VHL) ubiquitination pathway. However, it is unknown whether PHD and VHL exert their enzymatic activities on HIF-1α separately or as a multiprotein complex. Here, we show that phospholipase D1 (PLD1) protein itself acts as a molecular platform, interacting directly with HIF-1α, PHD, and VHL, thereby dynamically assembling a multiprotein complex that mediates efficient degradation of HIF-1α in an O_2_-dependent manner. PLD1 depletion prevents degradation of HIF-1α; however, overall, PLD1 activity is predominantly involved in the upregulation of HIF-1α through increased translation, despite negative regulation of HIF-1α stability by PLD1 protein itself, suggesting dual roles of PLD1 in the regulation of HIF-1α. Disruption of the interactions of PLD1 with the proteins might be involved in hypoxic stabilization of HIF-1α. Interestingly, the pleckstrin homology domain interacting with these proteins promoted degradation of HIF-1α independent of oxygen concentration and suppressed tumor progression. These observations define a novel function of PLD1 as a previously unrecognized HIF-1α regulator.

## INTRODUCTION

There are two mammalian isoforms of phospholipase D (PLD), PLD1 and PLD2. These genes have several conserved regions including the phox (PX) and pleckstrin homology (PH) domains, as well as two catalytic regions (HKD motifs) [1]. PLD catalyzes the hydrolysis of phospholipid to phosphatidic acid (PA), a lipid secondary messenger. PLD participates in a variety of cellular functions, including cell proliferation, survival, vesicle trafficking, cytoskeletal reorganization, differentiation, and morphogenesis [2-4]. These functions are primarily mediated by the metabolic product, PA. There is a growing body of evidence that PLD protein plays crucial roles in regulation of biological functions of PLD through interaction with signaling biomolecules independent of lipase activity. PLD has a complex network that consists of many binding partners and can merge upstream signals via these interactions that delicately regulate its activity. The interrelationships between PLD and its binding partners enable it to act as a scaffold protein to increase signaling efficiency, integrate and coordinate complex upstream signals, determine which signals will be transmitted to downstream pathways, and then amplify downstream signals [1]. In fact, the PX domain of PLD2 acts as both a GTPase activating protein (GAP) for dynamin and a potent guanine nucleotide exchange factor (GEF) for many small GTPases, regardless of its activity [5].

Hypoxia-inducible factor 1 (HIF-1) is a heterodimeric transcription factor consisting of HIF-1α and HIF-1β subunits that plays a central role in cellular adaptation to changes in oxygen availability [6]. While HIF-1β is constitutively expressed, HIF-1α is post-translationally regulated, thereby acting as a determinant of HIF-1 activity. Under normoxia, the HIF-1α is rapidly degraded via the ubiquitin-proteasome pathway through an E3 ubiquitin ligase, the von Hippel-Lindau (VHL) tumor suppressor gene product (VHL) [7]. VHL recognition of HIF-1α in the proteasomal destruction of HIF-1α protein occurs via hydroxylation of the two proline residues (Pro402 and Pro564) within the oxygen dependent degradation (ODD) domain in HIF-1α through HIF-1-prolyl hydroxylases (PHD) [8, 9]. Under hypoxic conditions, the enzymatic activities of PHDs are inhibited, preventing hydroxylation of HIF-1α, which results in its escape from VHL recognition and subsequent ubiquitination. This leads to HIF-1α stabilization accompanied by its nuclear translocation, heterodimerization with HIF-1β, and transcription of genes involved in angiogenesis, cell survival and proliferation [10-12]. VHL-dependent degradation of HIF-1α is influenced by multiple proteins that are engaged in various biological functions, suggesting cross-talk between the HIF pathway and numerous signaling pathways. In fact, OS-9 [13], spermidine/spermine*N*-acetyltransferase 2 (SSAT2) [14] and minichromosome maintenance (MCM) protein-7 regulate the stability of HIF-1α protein by intervening in the canonical pathway [15]. The LIM domain-containing protein1 (LIMD1), which mediates assembly of a PHD2-LIMD1-VHL protein complex to facilitate degradation of HIF-1α [16], was recently added to such proteins.

PLD activity in renal cancer cells lacking VHL was recently reported to be involved in the expression of HIF [17]. During elaboration of PLD1-mediated HIF-1α regulation, we found that, while the enzymatic activity of PLD1 is responsible for the increased level of HIF-1α via promotion of translation, PLD1 protein itself destabilizes HIF-1α protein by interacting directly with the components involved in VHL-dependent degradation of HIF-1α, independent of PLD activity. This study provides evidence that PLD1 protein functions as a platform molecule facilitating the dynamic assembly of PHD-HIF-1α-VHL, thereby mediating efficient degradation of HIF-1α through an oxygen-dependent pathway.

## RESULTS

### PLD1 plays a dual role in regulation of the cellular level of HIF-1α protein

**Figure 1:**
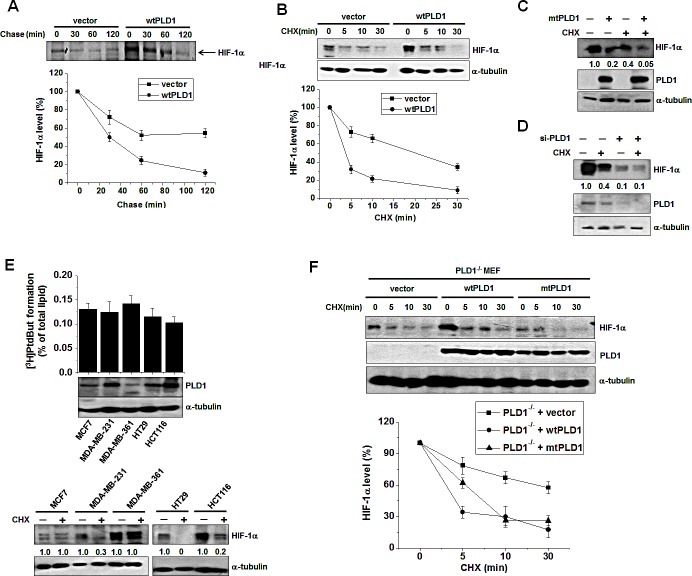
PLD1 plays a dual role in regulation of the cellular level of HIF-1α protein (A) Pulse-chase assay of HIF-1α in HEK293 cells transfected with vector or wtPLD1. The cells were pulse labeled with [^35^S]methionine-cysteinefor 4 h in the presence of MG132 and then chased in unlabeled medium for the indicated time. Lysates were immunoprecipitated with antibody to HIF-1α and assessed by autoradiography, after which the band intensity was quantified relative to the level of HIF-1α of no chase. Data are representative of three independent experiments. (B) Effects of PLD1 on the stability of HIF-1α. HEK293 cells were transfected with vector or PLD1 and then incubated under hypoxia (1% O_2_) for 4 h. The cells were subsequently reoxygenated (21% O_2_) and treated in parallel with CHX (100 μg/ml) for the indicated time. Lysates were analyzed by immunoblotting, after which the band intensity was quantified. The levels of HIF-1α to α-tubulin were normalized. Data are representative of three independent experiments. (C) HEK293 cells were transfected with mtPLD1 and incubated under hypoxia for 4 h, then reoxygenated by treatment with CHX for 30 min. Lysates were subsequentlyimmunoblotted using the indicated antibodies, after which the band intensity was quantified and the levels of HIF-1α to α-tubulin were normalized. Data are representative of three independent experiments. (D) HEK293 cells were transfected with PLD1 siRNA and incubated under hypoxia. The cells were then reoxygenated and in parallel and treated with CHX for 30 min, after which lysates were immunoblotted using the indicated antibodies. The band intensity was quantified and the levels of HIF-1α to α-tubulin were normalized. Data are representative of threeindependent experiments. (E) PLD activity assay and immunoblot analysis. Various cancer cells were incubated under hypoxia and then reoxygenated in parallel with treatment of CHX for 10 min. Lysates were subsequently analyzed by immunoblotting and the band intensity was quantified, after which the levels of HIF-1α to α-tubulin were normalized. Data are representative of three independent experiments. (F) PLD1-null MEF were transfected with the indicated constructs and then treated with CHX (100 μg/ml) for the indicated time. Lysates were immunoblotted with the indicated antibody. The band intensity of HIF-1α was quantified and the levels of HIF-1α to α-tubulin were normalized. Data are representative of three independent experiments.

A previous study showed that 1-butanol, but not tertiary butanol, suppressed HIFα expression in VHL-deficient renal cancer cells [17]. Primary alcohol blocks PLD-hydrolyzed PA formation by competing with water as a nucleophile, causing the formation of phosphatidylalcohol in a transphosphatidylation reaction; however, it does not fully block PA formation [4]. Thus, it is not clear whether PLD activity is required for expression of HIF-1α. We found that the PLD inhibitor, 5-fluoro-2-indolyl des-chlorohalopemide (FIPI), reduced the expression of HIF-1α, with a 1 h pulse resulting in an approximately 3 fold decrease, and a 2 h pulse leading to an approximately 2.65 fold decrease at the translational level when protein synthesis was measured by a [^35^S] methionine pulse assay (Figure S1A). These findings indicate that the catalytic activity of PLD1 regulates the translation rate of HIF-1α. However, the PLD inhibitor had no effect on the mRNA level of HIF-1α, and both wild type (wt) and catalytically inactive mutant (mt) PLD1 showed results comparable to that of PLD inhibitor (see also Figure S1B), suggesting that PLD1 activity does not affect expression of HIF-1α at the transcriptional level. Overexpression of wtPLD1 increased the expression of endogenous HIF-1α protein, but mtPLD1 decreased the protein level of basal HIF-1α relative to that of vector (see also Figure S1C). Thus, it is likely that PLD1 activity is predominantly involved in the expression of HIF-1α through increased translation. To further examine the effects of PLD1 on the stability of HIF-1α, we conducted pulse-chase experiments. In these experiments, HEK293 cells were transfected with vector or PLD1 and then labeled with [^35^S] methionine/cysteine, after which they were incubated (chased) for the indicated time in normal media. Immunoprecipitated HIF-1α was detected by autoradiography and quantified by densitometry. Surprisingly, PLD1 decreased the stability of HIF-1α relative to that of vector (from a HIF-1α half-life of 60 min to 30 min) (Figure 1A). HIF-1α protein is detected at very low levels under normoxia; thus, cells were switched from hypoxia to normoxia (reoxygenation) to enable easy detection of the change in HIF-1α protein. The reoxygenated cells were also treated with cycloheximide (CHX) to block new protein synthesis. WtPLD1 enhanced degradation of endogenous HIF-1α protein from a half-life of approximately 22 min to 3 min (Figure 1B). MtPLD1 also accelerated degradation of HIF-1α protein (Figure 1C and S1D), suggesting that PLD1 decreases the stability of HIF-1α protein, independent of its lipase activity. We further examined the effects of catalytically inactive PLD1 on translation. PLD1 mutant dramatically decreased the expression of HIF-1α at the translational level when analyzed by pulse assay (see Figure S1E), indicating the involvement of PLD1 activity in the translation of HIF-1α. To further confirm PLD1-mediated destabilization of HIF-1α, we examined whether knockdown of PLD1 could delay degradation of HIF-1α protein. To accomplish this, cells transfected with PLD1 siRNA or control siRNA were subjected to hypoxia and subsequent reoxygenation, after which the HIF-1α levels were monitored. PLD1 depletion significantly decreased the PLD activity (see also Figure S1F). PLD1 siRNA greatly decreased endogenous HIF-1α protein levels when compared to treatment with scrambled siRNA (Figure 1D), which was likely caused by the translational inhibition of HIF-1α due to reduced PLD activity. Simultaneously, HIF-1α degradation was clearly prevented by PLD1 knockdown (lane 3 vs. lane 4) (Figure 1D). We also found that both wt and mtPLD1 accelerated degradation of HIF-2α protein (see also Figure S1G). To further examine whether the HIF-1α destabilizing effect was elicited by endogenous PLD1 protein, we investigated breast cancer cells and colorectal cancer cells, which express different levels of PLD1 protein but show similar levels of PLD activity, enabling the possibility of PLD activity in such an effect to be excluded. PLD activity is not necessarily due to expression of PLD protein [17]. Although basal activity of PLD can largely be derived from PLD2, PLD1 protein was identified as a major isoform of PLD in these cells. Thus, it is speculated that the basal activity of PLD in the cells may predominantly be due to PLD1 activity. However, the possibility that a very small amount of PLD2 protein in those cells might also contribute to the basal PLD activity, cannot be excluded. Cells expressing high levels of PLD1 protein (MDA-MB-231, HT29 and HCT116) showed rapid degradation of endogenous HIF-1α in the presence of CHX when compared to those with low levels of PLD1 protein (MCF7 and MDA-MB-361) (Figure 1E). Furthermore, we examined the physiological relevance in PLD1-deficient mouse embryonic fibroblasts (MEF) to confirm these findings. When PLD1-deficient MEF were transfected with wt or mtPLD1, PLD1 decreased the stability of HIF-1α independent of its lipase activity (Figure 1F). Collectively, our data demonstrate that, in addition to enhancing translation of HIF-1α, PLD1 functions to negatively regulate HIF-1α stability, regardless of its enzymatic activity.

### PLD1 directly interacts with HIF-1α and PHD2 and promotes prolyl hydroxylation of HIF-1α

**Figure 2:**
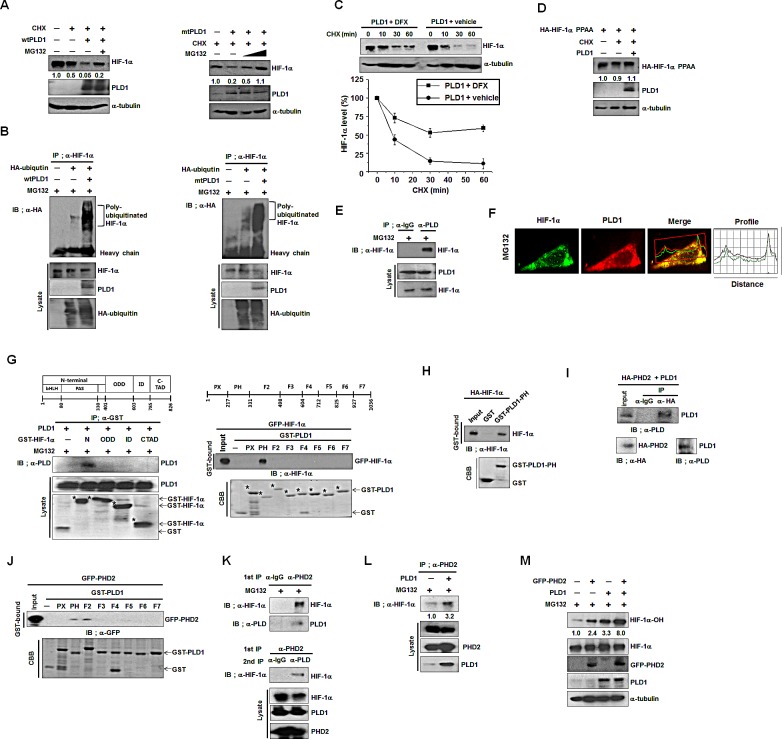
PLD1 directly interacts with HIF-1α and PHD2 and promotes prolyl hydroxylation of HIF-1α (A) The effect of MG132 on the level of endogenous HIF-1α. HEK293 cells were transfected with wt or mtPLD1 and incubated under reoxygenation conditions while treated in parallel with CHX and/or MG132 for 30 min. Lysates were analyzed by immunoblotting, after which the band intensity was quantified. The levels of HIF-1α to α-tubulin were normalized. Data are representative of three independent experiments. (B) Immunoprecipitation (IP) assay was performed to measure the ubiquitination of endogenous HIF-1α from HEK293 cells that were cotransfected with the indicated constructs in the presence of MG132. Data are representative of three independent experiments. (C) Effects ofthe PHD inhibitor, DFX (100μM), on the stability of HIF-1α regulated byPLD1 under reoxygenation conditions. Lysates were analyzed by immunoblotting, after which the band intensity was quantified. The levels of HIF-1α to α-tubulin were normalized. Data are representative of three independent experiments. (D) Effects of PLD1 on the stability of HIF-1α-PPAA, which contains the proline-to-alanine substitutions, P402A/P564A. Lysates were analyzed by immunoblotting and the band intensity was quantified. The levels of HIF-1α to α-tubulin were normalized. Data are representative of three independent experiments. (E) IP assay was performed to study the interaction of endogenous HIF-1α with PLD1 in the presence of MG132. Data are representative of three independent experiments. (F) HEK293 cells were pretreated with MG132 for4 h. The colocalization between HIF-1α and PLD1 was analyzed. Representative fluorescence microphotographs are shown together with theprofiles of colocalization. (G) IP assay and GST pull-down assay were performed to determine the binding domain mapping of HIF-1α (left) and PLD1 (right). N, N-terminal region (1 - 401 residues) containing the basic helix–loop-helix (bHLH)/PER–ARNT–SIM (PAS); ODD, oxygen dependent degradation domain (401 - 603 residues); ID, inhibitory domain (576-785 residues); CTAD, C-terminal transactivation domain (786 - 826 residues). Data are representative of three independent experiments. (H) GST pull-down assay of *in vitro* translated-HA-HIF-1α and purified GST-PLD1-PH. Data are representative of three independent experiments. (I) IP assay of *in vitro*translated-HA-PHD2 and PLD1. Data are representative of three independent experiments.(J) GST pull-down assay of the binding domain mapping of PLD1 interacting with PHD2. Data are representative of three independent experiments. (K) CoIP assay of lysates prepared from HEK293 cells in the presence of MG132. Lysates were immunoprecipitated with anti-PHD2 antibody and immunoblotted with the indicated antibody. The proteins released from primary immunoprecipitates were reimmunoprecipitated with antibody to PLD and analyzed by immunoblot with anti-HIF-1α. Data are representative of three independent experiments. (L) Effect of PLD1 on the interaction of PHD2 with HIF-1α in the presence of MG132. Data are representative of three independent experiments. (M) Effects of PLD1 on the hydroxylation of HIF-1α. HEK293 cells were cotransfected with the indicated constructs in the presence of MG132. Lysates were analyzed by immunoblotting, after which the band intensity was quantified. The levels of hydroxylated HIF-1α to total HIF-1α were normalized. Data are representative of three independent experiments.

Since PLD1 protein itself destabilizes HIF-1α protein, we examined whether PLD1-mediated degradation of HIF-1α occurred via the ubiquitin-proteasome pathway, which is the main destructive pathway for HIF-1α. As shown in Figure 2A, degradation of endogenous HIF-1α protein mediated by both wtPLD1 and mtPLD1 was greatly prevented by the proteasome inhibitor, MG132 (Figure 2A). Moreover, both wtPLD1 and mtPLD1 showed increased levels of ubiquitination of endogenous HIF-1α (Figure 2B). Proteasomal degradation of HIF-1α is initiated by HIF-1α hydroxylation through PHD, leading to VHL-dependent ubiquitination and proteasomal degradation. We examined whether HIF-1α degradation by PLD1 protein required hydroxylation of HIF-1α. To accomplish this, HIF-1α destabilization by PLD1 was monitored in the presence of desferrioxamine (DFX), an inhibitor of PHD. DFX substantially delayed PLD1-mediated degradation of HIF-1α (Figure 2C). To further examine the hydroxylation requirement, we utilized HIF-1α-PPAA (an HIF-1α mutant resistant to hydroxylation-dependent degradation), which contains the proline-to-alanine substitutions, P402A/P564A. As shown in Figure 2D, PLD1 showed no destabilizing effect on HIF-1α-PPAA protein, indicating that PHD-dependent hydroxylation is involved in PLD1-mediated HIF-1α degradation. The stability and activity of HIF-1α are regulated by physical association with various proteins [18]. To investigate whether there is a physical interaction among PLD1, HIF-1α and PHD, we first examined the interaction between HIF-1α and PLD1 using coimmunoprecipitation. PLD1 associated with endogenous HIF-1α, as well as with exogeneous HIF-1α (Figure 2E, see also Figure S2A). In addition, mtPLD1 interacted with HIF-1α (see also Figure S2B). Furthermore, endogenous HIF-1α was colocalized with endogenous PLD1 in the presence of MG132 under normoxia, suggesting a dynamic physiological interaction of the proteins (Figure 2F). To identify protein domains in which there was a mutual interaction between PLD1 and HIF-1α, an immunoprecipitation assay was performed using a panel of mammalian expressed GST-HIF-1α fusion proteins or GST-PLD1 fusion proteins. PLD1 bound specifically to the GST-HIF-1α-N-terminal domain (1-401 residues) (Figure 2G, left). Similarly, the PH domain (217-331) of PLD1 was found to interact with HIF-1α (Figure 2G, right). To verify that the interaction of PLD1 with HIF-1α was direct, we conducted an in vitro binding assay using the purified GST-PLD1-PH fusion proteins and *in vitro*-translated HA-HIF-1α. HIF-1α bound GST-PLD1-PH domain, but not GST alone (Figure 2H), indicating a direct interaction between HIF-1α and PLD1. We next examined whether PLD1 was associated with PHD. Since PHD2, one of the PHD family members, is the main oxygen sensor responsible for regulation of HIF-1α [19, 20], this hydroxylase was used for subsequent experiments. Our data showed that both wtPLD1 and mtPLD1 interacted with PHD2 (Figure S2C, S2D). We also verified that the interaction between PLD1 and PHD2 was direct, as shown by *in vitro* binding assays using *in vitro-*translated PLD1 and PHD2 (Figure 2I). To determine which regions of PLD1 mediate its interaction with PHD2, we conducted a GST-pull down assay using a panel of GST-PLD1 fragments. The results revealed that PHD2 bound to the PH domain and F2 region (331-497) of PLD1 (Figure 2J). Furthermore, endogenous PHD2 immunoprecipitated endogenous HIF-1α and PLD1, while PLD1 in the eluent from the PHD2 immune-complexes coimmunoprecipitated HIF-1α Figure 2K), suggesting the existence of a HIF-1α-PLD1-PHD2 triple complex. Finally, we examined whether HIF-1α interacted with PHD2, and if so, if this interaction was affected by PLD1. HIF-1α was associated with PHD2, while wtPLD1 and mtPLD1 increased the association of HIF-1α with PHD2 (Figure 2L, see also Figure S2E). These findings prompted us to explore whether PLD1 promotes PHD2-mediated hydroxylation of HIF-1α. HEK293 cells were transfected with PLD1 and/or PHD2 and then treated with MG132 while monitoring the levels of hydroxylated HIF-1α. As shown in Fig 2M, PLD1 or PHD2 promoted hydroxylation of HIF-1α, while coexpression of PLD1 with PHD2 synergistically enhanced the hydroxylation of HIF-1α. mtPLD1 also increased hydroxylation of HIF-1α, and this effect was potentiated by coexpression with PHD2 (see also Figure S2F). Taken together, these findings suggest that PLD1 associates with both HIF-1α and PHD2 to form a triple complex, promoting polylhydroxylation of HIF-1α via enhancement of the PHD2-HIF-1α interaction, independent of the lipase activity.

### PLD1 enhances VHL-dependent HIF-1α degradation by accelerating the association between VHL and HIF-1α

**Figure 3:**
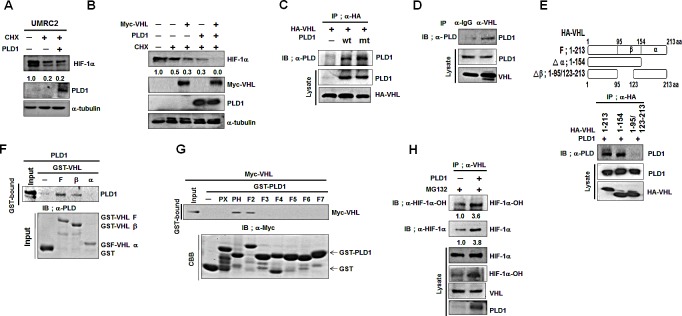
PLD1 enhances VHL-dependent HIF-1α degradation by accelerating the association between VHL and HIF-1α (A) Effect of PLD1 on the stability of HIF-1α in VHL-deficient UMRC cells. Lysates were analyzed by immunoblot and the band intensity was quantified. The levels of HIF-1α to α-tubulin were normalized. Data are representative of three independent experiments. (B) Effect of PLD1 on VHL-mediated endogenous HIF-1α degradation in the presence of CHX. Lysates were analyzed by immunoblot and the band intensity was quantified. The levels of HIF-1α to α-tubulin were normalized. Data are representative of three independent experiments. (C) IP assay of lysatesof HEK293 cells cotransfected with HA-VHL and wtPLD1 or mtPLD1. Data are representative of three independent experiments. (D) IP assay for the interaction of VHL with PLD1. Data are representative of three independent experiments. (E) IP assay for the binding domain mapping of HA-VHL interacting with PLD1. Data are representative of three independent experiments.(F) GST pull-down assay of *in vitro*translated-PLD1 and GST-VHL fragments. Data are representative of threeindependent experiments. (G) GST pull-down assay for the binding domainmapping of PLD1 interacting with VHL. Data are representative of three independent experiments. (H) Effect of PLD1 on the interaction of VHL with hydroxylated HIF-1α in the presence of MG132. The band intensity was quantified and the ratios of hydroxylated HIF-1α to HIF-1α were normalized. Data are representative of three independent experiments.

PHD hydroxylation of HIF-1α enables an E3 ubiquitin ligase, VHL, to bind to and destroy HIF-1α via the proteasomal pathway. We investigated whether PLD1 was involved in this process. To accomplish this, the requirements of VHL were examined in PLD1-mediated HIF-1α degradation. PLD1 did not destabilize HIF-1α in VHL-deficient UMRC cells (Figure 3A). We further examined whether PLD1 modulated VHL-mediated HIF-1α degradation. Transfection with PLD1 or VHL accelerated degradation of endogenous HIF-1α, which was synergized by coexpression of PLD1 with VHL (Figure 3B). These data suggest that PLD1 cooperates with VHL to enhance VHL-mediated HIF-1α degradation. To investigate how this occurs, we examined whether PLD1 interacts with VHL. Interestingly, VHL immunoprecipitated with both wt and mtPLD1 (Figure 3C), and VHL was associated with endogenous PLD1 (Figure 3D). As previously reported [21], endogenous HIF-1α was coimmunoprecipitated with VHL and Elongin C, a component of E3 ubiquitin ligase (see also Figure S3A). Coimmunoprecipitation and GST-pull down assay using fragments of GST-VHL and *in vitro*-translated PLD1 showed that PLD1 interacted directly with the β-domain of VHL (Figure 3E, F). Similarly, GST-pull-down assay using various GST-PLD1 fragments revealed that VHL bound to the PH domain and F2 domain of PLD1 (Figure 3G). Although we demonstrated the association of PLD1 with VHL, it was still not clear how PLD1 and VHL cooperate to degrade HIF-1α. Since hydroxylation of HIF-1α, which was enhanced by PLD1, is critical to the HIF-1α-VHL interaction [22, 23] and PLD1 binds to PHD2, VHL and HIF-1α through its PH domain, PLD1 may provide a microenvironment in which VHL binding to HIF-1α occurs effectively, resulting in promotion of HIF-1α degradation. To test this, we examined whether PLD1 affected the interaction of VHL with HIF-1α. WtPLD1 and mtPLD1 enhanced the interaction of VHL with hydroxylated HIF-1α (Figure 3H, see also Figure S3B). Taken together, these findings suggest that PLD1 enhances VHL-dependent HIF-1α degradation, probably by accelerating the association between VHL and HIF-1α.

### Hydroxylation of HIF-1α drives dissociation of HIF-1α-VHL complex from PLD1

**Figure 4:**
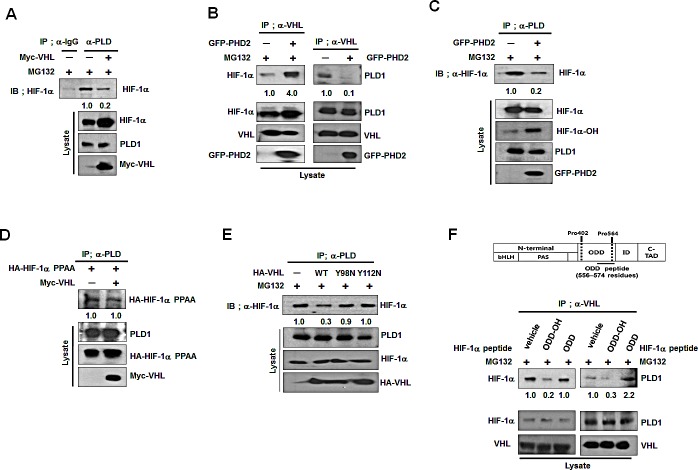
Hydroxylation of HIF-1α drives dissociation of HIF-1α-VHL complex from PLD1 (A) Effect of VHL on the interaction of PLD1 with HIF-1α in the presence ofMG132. The band intensity was quantified. Data are representative of three independent experiments. (B) Effect of PHD2 on the interaction of VHL with PLD1. Data are representative of three independent experiments.(C) Effect of PHD2 on the interaction of PLD1 with HIF-1α in the presence of MG132. The band intensity was quantified. Data are representative of three independent experiments. (D) Effect of VHL on the interaction of PLD1 with HIF-1α-PPAA. The band intensity was quantified. Data are representative of three independent experiments. (E) IP assay to test the effects of mutants of VHL Y98N and Y112N on theinteraction of PLD1 with HIF-1α in the presence of MG132. The band intensity was quantified. Data are representative of three independent experiments. (F) IP assay to test the effects of hydroxylated HIF-1α peptide (residues 556-574) on the interaction of VHL with HIF-1α and PLD1 in the presence of MG132. ODD-OH, hydroxylated-ODD peptide; ODD, nonhydroxylated-ODD peptide. The band intensity was quantified. Data arerepresentative of three independent experiments.

PLD1 binds to the β-domain of VHL, interacting with HIF-1α, and the VHL-HIF-1α interaction is initiated and strengthened by hydroxylation of HIF-1α. Thus, hydroxylated HIF-1α may become a competitor of PLD1 binding to VHL. To test this possibility, we examined whether VHL and HIF-1α competed for association with PLD1. Ectopic expression of VHL substantially attenuated interaction of PLD1 with HIF-1α (Figure 4A). In addition, transfection with HIF-1α reduced the level of PLD1 in the immune-complex of VHL, while VHL effectively bound to hydroxylated HIF-1α (see also Figure S4A). To further investigate this process, PHD2 was cotransfected and changes in mutual interactions among PLD, HIF-1α and VHL were monitored. We expected PHD2 to promote interaction of HIF-1α with VHL while attenuating interaction between PLD1 and either HIF-1α or VHL. As predicted, PHD2 increased interaction of HIF-1α with VHL, but the association of both PLD1-HIF-1α and PLD1-VHL decreased (Figure 4B and 4C). Moreover, inhibition of HIF-1α hydroxylation by DFX increased interaction of PLD1 with HIF-1α (see also Figure S4B), which was inhibited by PHD2 overexpression and greatly recovered by DFX (see also Figure S4C). To confirm these findings, the protein interactions among PLD1, HIF-1α and VHL were examined following transfection with HIF-1α-PPAA or VHL Y98N and Y112N, which are VHL mutants incapable of binding to hydroxylated HIF-1α [24]. VHL did not affect association between PLD1 and mutant HIF-1α (Figure 4D). Unlike wtVHL, VHL Y98N and Y112N failed to reduce the interaction of PLD1 with HIF-1α (Figure 4E). In addition, hydroxylated HIF-1α peptide corresponding to the 556-574 residues of HIF-1α, abolished the association of VHL with PLD1 when applied to cell lysates (Figure 4F). Conversely, nonhydroxylated HIF-1α peptide had no effect on this association (Figure 4F). As a control, hydroxylated HIF-1α peptide, but not nonhydroxylated HIF-1α peptide, reduced association of VHL with HIF-1α. These data strongly suggest that hydroxylation of HIF-1α favors interaction with VHL over PLD1, which drives dissociation of the VHL-HIF-1α complex from PLD1.

### Protein interactions of PLD1 with HIF-1α, PHD2 and VHL are abolished under hypoxia

**Figure 5:**
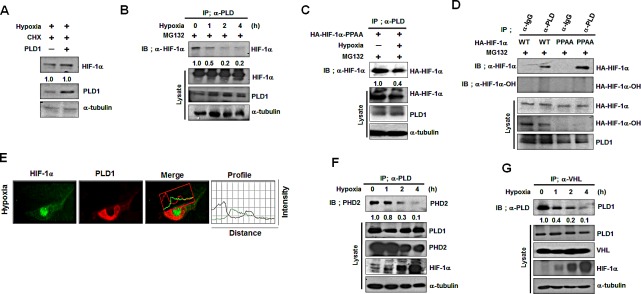
The protein interactions of PLD1 with HIF-1α, PHD2 and VHL are abolished under hypoxia (A) Effect of PLD1 on the stability of HIF-1α under hypoxia. Lysates were analyzed by immune blot. The band intensity was quantified and the levels of HIF-1α to α-tubulin were normalized. Data are representative of three independent experiments. (B) IP assay for the interaction of PLD1 with HIF-1α under hypoxic conditions. The band intensity was quantified. Data are representative of three independent experiments. (C) IP assay was performed to test the effects of hypoxia on the interaction of PLD1 with HA-HIF-1α-PPAA in the presence of MG132. The band intensity was quantified. Data are representative of three independent experiments. (D) IP assay of lysates prepared from HEK293 cells that were cotransfected with HA-HIF-1α (wild type or PPAA mutant) and PLD1 in the presence of MG132. The band intensity was quantified. Data are representative of three independent experiments. (E) HEK293 cells were incubated under hypoxic conditions, after which colocalization between HIF-1α and PLD1 was analyzed. Representative fluorescence microphotographs are shown together with the profiles of colocalization. Data are representative of three independent experiments. (F) Effect of hypoxia on the interaction of PLD1 with PHD2. The band intensity was quantified. Data are representative of three independent experiments. (G) Effect of hypoxia on the interaction of VHLwith PLD1. The band intensity was quantified. Data are representative of three independent experiments.

Our data demonstrate that PLD1 is a previously unrecognized component in the canonical HIF-1α degradation pathway, which is responsive to oxygen concentration. Thus, PLD1 regulation of HIF-1α stability is likely influenced by oxygen concentration. To test this, PLD1 was overexpressed under hypoxia and the stability of HIF-1α was examined. Unlike normoxia, PLD1 did not affect the stability of endogenous and exogenous HIF-1α under hypoxic conditions (Figure 5A, see also Figure S5A). We next investigated whether hypoxia affected the mutual interactions among proteins. To accomplish this, HEK293 cells were cultured under hypoxic conditions, after which the interactions were examined by immunoprecipitation. Hypoxia abolished the interaction of PLD1 with HIF-1α (Figure 5B). This hypoxic effect occurred regardless of the state of hydroxylation of HIF-1α since association of PLD1 with HIF-1α-PPAA was also suppressed under hypoxia (Figure 5C), while HIF-1α-PPAA effectively associated with PLD1 under normoxia (Figure 5D). These findings suggest that oxygen is involved in disruption of the interaction of PLD1 with HIF-1α. Moreover, endogenous PLD1 was not colocalized with endogenous HIF-1α under hypoxia (Figure 5E), and the interaction of PLD1 with either PHD2 or VHL was suppressed (Figure 5E, F). As previously reported [23], the interaction between VHL and HIF-1α was reduced under hypoxic conditions (see also Figure S5B). Taken together, these results indicate that, unlike normoxia, hypoxic conditions abolish the protein interactions of PLD1 with HIF-1α, PHD2 and VHL.

### Disruption of the interaction of PLD1 with HIF-1α might be involved in hypoxic stabilization of HIF-1α

**Figure 6:**
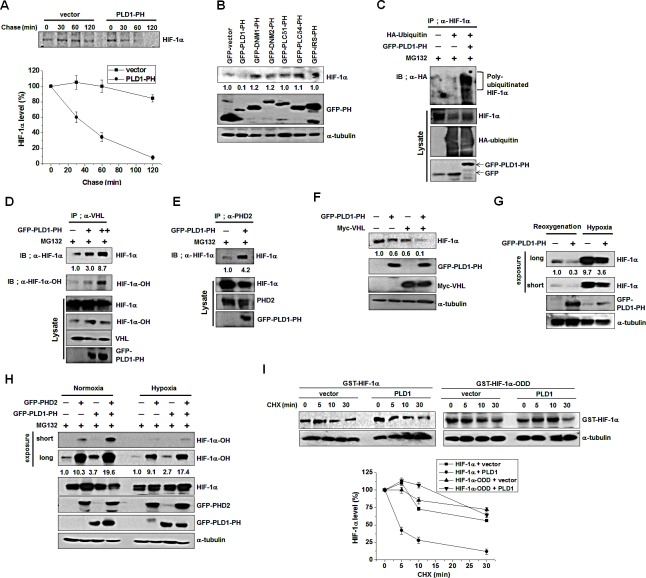
Disruption of the interaction of PLD1 with HIF-1α contributes to hypoxic stabilization of HIF-1α (A) Pulse-chase assay of HIF-1α in HEK293 cells transfected with PLD1-PH. Lysates were immunoprecipitated with antibody to HIF-1α and assessed by autoradiography, followed by quantification of the band intensity. Data are representative of three independent experiments. (B) Effect of various PH domains on the expression of HIF-1α. HEK293 cells were transfected with GFP-PH domain of various proteins and then reoxygenated. Lysates were analyzed by immunoblotting, after which the band intensity was quantified and the levels of HIF-1α to GFP-PH were normalized. Data are representative of three independent experiments. (C) Effect of PLD1-PH on the ubiquitination of HIF-1α in the presence ofMG132. The band intensity was quantified. Data are representative of three independent experiments. (D) Effect of PLD1-PH on the interaction of VHL with HIF-1α in the presence of MG132. The band intensity was quantified. Data are representative of three independent experiments. (E) Effect of PLD1-PH on the interaction of PHD2 with HIF-1α in the presence of MG132. Data are representative of three independent experiments. (F) HEK293 cells were cotransfected with PLD1-PH and/or VHLand then reoxygenated. The level of HIF-1α was analyzed by immune blot. The levels of HIF-1α to α-tubulin were quantified and normalized. Data are representative of three independent experiments. (G) IB analysis of endogenous HIF-1α by GFP-PLD1-PH under reoxygenation and hypoxia conditions. The levels of HIF-1α to α-tubulin were quantified and normalized. Data are representative of three independent experiments. (H) IB analysis of hydroxylated HIF-1α of lysates from HEK293 cells cotransfected with PHD2 and/or GFP-PLD1-PH under normoxia and hypoxia conditions in the presence of MG132. The levels of hydroxylated HIF-1α to HIF-1α were quantified and normalized. Data are representative of three independent experiments. (I) Effect of PLD1 on the stability of HIF-1α-ODD. HEK293 cells were cotransfected with the indicated constructs. CHX was added for the indicated time and lysates were analyzed by immunoblotting, after which the band intensity was quantified. The levels of HIF-1α to α-tubulin were quantified and normalized. Data are representative of three independent experiments.

HIF-1α is still degraded under hypoxia via the degradative pathway in which PHD and VHL are engaged [25-28]. In fact, LIMD, which is associated with VHL and PHD regardless of oxygen concentration, remains functional as a molecular scaffold for efficient degradation of HIF-1α through the canonical pathway under hypoxia [16]. These findings suggest that hypoxic stabilization of HIF-1α may be ascribed to disruption of the interactions of PLD1 with proteins including HIF-1α in addition to inhibition of PHD2. To test this scenario, we first examined whether the PH domain of PLD1 (PLD-PH) itself, which binds to HIF-1α, PHD and VHL, could act as a regulator of HIF-1α stability. Pulse-chase experiments were conducted in HEK293 cells transfected with empty vector or PLD1-PH. Similar to full length PLD1, PLD1-PH promoted destabilization of HIF-1α relative to vector (Figure 6A). We further examined whether the PH domains in other proteins share the ability of PLD-PH to degrade HIF-1α. With the exception of r PLD1-PH, the PH domains of dynamin 1 (DNM1), dynamin 2 (DNM2), phospholipase C-δ1(PLCδ1), phospholipase C-δ4 (PLCδ4), and insulin receptor substrate (IRS) had no ability to interact with and reduce HIF-1α (Figure 6B, see also Figure S6A). To demonstrate that PLD1-PH exerts its destabilizing effect through the same mechanism as intact PLD1, a series of experiments were repeated with PLD1-PH. As demonstrated by intact PLD1, PLD1-PH promoted prolyl hydroxylation of HIF-1α and ubiquitination of endogenous and exogenous HIF-1α (Figure 6C, see also Figure S6B). Moreover, PLD1-PH promoted interaction of HIF-1α with VHL and PHD2 (Figure 6D, E). PLD1-PH or VHL decreased the level of HIF-1α protein, while cotransfection with VHL and PLD1-PH synergistically reduced the endogenous HIF-1α protein level (Figure 6F), indicating that PLD1-PH mechanistically acts as intact PLD under normoxia. To determine whether PLD-PH exhibited the same biological properties under hypoxia, PLD1-PH was subjected to the same experiments under hypoxia. Surprisingly, PLD1-PH still decreased the level of HIF-1α protein under hypoxia (Figure 6G), which is in stark contrast with intact PLD1. Furthermore, PLD1-PH or PHD2 promoted hydroxylation of HIF-1α under both normoxic and hypoxic conditions, while coexpression of PLD1-PH with PHD2 enhanced the hydroxylation of HIF-1α relative to that of either expression under both conditions (Figure 6H). To examine the relevance of these findings, we investigated the interaction of PLD-PH with HIF-1α, PHD and VHL. As expected, the interactions of PLD1-PH with HIF-1α, PHD2, and VHL were maintained under both normoxia and hypoxia (see also Figure S6C-E), and PLD1-PH still promoted interaction of HIF-1α with VHL (Figure S6F). These findings indicate that PLD1-PH forms complexes with these proteins and functions as intact PLD1 in an O_2_-independent manner. PLD1-PH-mediated degradation of HIF-1α occurs independently of PLD activity as PLD1-PH does not have lipase activity (see also Figure S6G). Additionally, PLD1-PH promoted degradation of HIF-2α (see also Figure S6H). To further test this process, we examined whether an ODD domain of HIF-1α (HIF-1α-ODD, 401-603 residues), which is susceptible to canonical degradation but does not bind to PLD1 (Figure 6I), elicited resistance to PLD1-mediated degradation. Although PLD1 significantly accelerated degradation of full length HIF-1α, ectopic expression of PLD1 did not increase the degradation rate of HIF-1α-ODD relative to that of vector transfected cells. These results support our assumption that hypoxia might stabilize HIF-1α by inhibiting PHD as well as disrupting the interaction of PLD with the proteins.

### PH domain of PLD1 abolishes tumor progression

**Figure 7:**
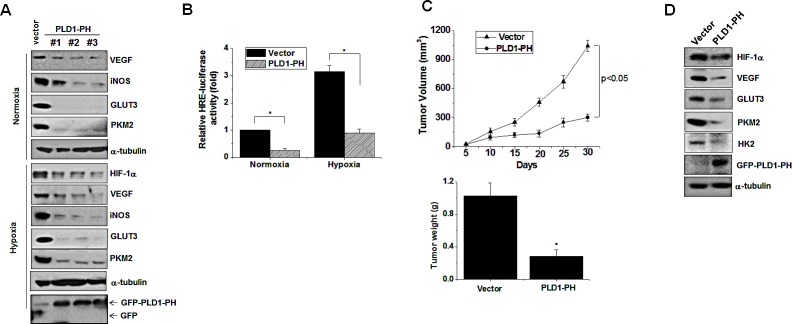
PLD1-PH suppresses tumor progression and expression of HIF-1α and its target genes (A) Immunoblot assay for expression of HIF-1α and its target genes under normoxia and hypoxia in PLD1-PH-expressed HT29 cells. Data are representative of three independent experiments. (B) Luciferase assay ofHRE under normoxia and hypoxia. * p<0.01. The values are the mean ± S.D. of three independent experiments. (C) The volume and weight of xenografted tumors were measured. Data were expressed as the mean ± S.D. of seven different mice. * p<0.05 (D) Immunoblot analysis of tumor tissues derived from the xenografted mice. Data are representative of three independent experiments.

Unlike intact PLD1, PLD1-PH itself promotes degradation of HIF-1α, even under hypoxia. The crucial role of HIF in tumorigenesis can be inferred from the observation that the level of HIFα is positively correlated with cancer progression in hypoxic microenvironments [29]. Thus, we examined the role of PLD1-PH in tumorigenesis. Three types of HC29 clones stably expressing PLD1-PH markedly decreased the expression of HIF-1α target genes (VEGF, iNOS, GLUT3, PKM2) under both normoxia and hypoxia (Figure 7A). Moreover, PLD1-PH significantly decreased transactivation of HIF-1α under normoxia and hypoxia (Figure 7B). The PLD1-PH also reduced proliferation of colon cancer cells relative to the control (Figure S7A). Furthermore, PLD1-PH led to significant tumor regression in tumor xenografts when compared to vector cells (Figure 7C). As expected, the PH domain led to a remarkable decrease in expression of HIF-1α and its target gene expression in tumor tissues (Figure 7D; see also Figure S7B). Taken together, these findings suggest a new role of the PH domain of PLD1 in tumor regression via targeting of HIF-1α.

## DISCUSSION

In this study, we demonstrate that PLD1 protein itself acts as a molecular platform, associating directly with HIF-1α, PHD2 and VHL, thereby assembling a multi-protein complex in which a dynamic process takes place between proteins, indicating that PLD1 protein represents a previously unrecognized hypoxic regulator. This molecular platform likely functions as a microenvironment for efficient post-translational modifications of HIF-1α, leading to degradation of the protein in an oxygen concentration-dependent manner. PLD activity in cells has been reported to be responsible for increased HIF-α protein levels [17]. The results of the present study show that PLD1 activity is required to enhance translation of HIF-α. Conversely, the PLD1 protein itself was found to shorten the half-life of the HIF-1α protein independent of PLD1 activity. The destabilizing effect of PLD1 was illustrated by a pulse and chase assay, and was further supported by our data demonstrating that depletion of PLD1 clearly delayed the degradation of endogenous HIF-1α, though PLD1 knockdown reduced the levels of HIF-1α due to diminished PLD1 activity. This novel notion of PLD1 function was extended to actual biological conditions by testing HIF-1α stability in a panel of cell lines with different levels of PLD1 protein and a similar level of PLD activity. The stability of HIF-1α was greatly decreased in cell lines with elevated levels of PLD1 relative to that of cells with relatively low levels of PLD1. However, the effect of PLD1 activity-induced HIF-1α translation appears to prevail over the destabilization effect of PLD1 protein itself, followed by predominant upregulation of HIF-1α, suggesting dual roles of PLD1 in the regulation of HIF-1α. We did not focus on mechanistic control of HIF-1α by PLD2 in this study, as our primary goal was investigation of PLD1. However, further studies to elucidate the regulatory mechanism of PLD2-mediated HIF-1α, are needed.

The canonical pathway for degradation of HIF-1α protein is initiated by PHD-mediated hydroxylation of HIF-1α. Hypoxia inactivates PHDs, causing accumulation of HIF-1α. In turn, HIF-1 further transactivates PHDs. It was suggested that the most relevant purpose of this feedback loop is to limit HIF-1α accumulation caused by growth factors and insulin under normoxia [30]. HIF-1α is regulated by two distinct mechanisms: by degradation and by translation. Under normoxia, HIF-1α is degraded via the classic PHD pathway, is expressed at low levels and therefore does not activate the feedback loop, except when HIF-1 is increased via mTOR-dependent translation [30]. But under hypoxia, HIF-1α transcriptionally activates its own degradation via at least two pathways one of which is independent from the PHD/VHL pathway [31].

Our data show that a PHD inhibitor and a HIF-1α mutation resistant to PHD-mediated hydroxylation prevented the PLD1 effect, indicating that PLD1-mediated degradation of HIF-1α requires HIF-1α hydroxylation. Moreover, PLD1 enhanced PHD hydroxylation of HIF-1α. We suggest that these effects result from PLD1 providing a binding site to bring HIF-1α and PHD close to each other, leading to effective hydroxylation of HIF-1α. In fact, PLD1 associates directly with both PHD and HIF-1α through its PH domain. Consistent with the enhanced PHD activity induced by PLD1, PLD1 increased physical association between PHD and HIF-1v. Hydroxylated HIF-1α is recognized by VHL, a ubiquitin ligase, leading to polyubiquitination and proteasomal degradation. Considering that PLD1-mediated degradation of HIF-1α was prevented by the proteasome inhibitor MG132, which accumulated ubiquitinylated HIF-1α, PLD1-mediated hydroxylation of HIF-1α should be linked to VHL dependent degradation. However, our data show that PLD1 does not accelerate HIF-1α degradation in VHL-defective cells. Moreover, transfection with VHL enhanced PLD1-mediated HIF-1α degradation, and synergetic degradation of HIF-1α was observed in response to cotransfection with PLD1 and VHL. Our findings suggest that participation of VHL in PLD-mediated HIF-1α degradation may occur through direct interaction with PLD1. Given that VHL binds to the PH domain of PLD1, where HIF-1α and PHD bind as well, it is possible that PLD1 plays a role as a reaction pot in which reactants react effectively with each other. Indeed, PLD1 promoted association of hydroxylated HIF-1α with VHL, as well as PHD hydroxylation of HIF-1α. Furthermore, our data show that PLD1 bound to the α domain of VHL, thereby sharing the binding site with hydroxylated HIF-1α. These findings imply that PLD1 acts as a molecular platform in which a dynamic process occurs between the components for HIF-1α degradation. In agreement with these findings, hydroxylation of HIF-1α appears to result in dissociation of the HIF-1α-VHL complex from PLD1, which likely occurs by displacing PLD1 with hydroxylated HIF-1α. These suggestions are based on compelling evidence provided by our data showing that (1) ectopic expression of VHL and HIF-1α compete with each other for association with PLD1, (2) PHD dissociates the VHL-PLD or HIF-1α-PLD1 interaction, while a PHD inhibitor increases the HIF-1α-PLD interaction, and (3) HIF-1α mutant resistant to hydroxylation or VHL mutants incapable of binding to hydroxylated HIF-1α did not compete with each other for PLD1 binding upon overexpression. It is likely that the regulatory role of PLD1 on HIF-1α stability is abolished under hypoxia. Considering that HIF-1α can still be degraded under hypoxia via the canonical pathway, such functional loss of hypoxic PLD1 is not in line with the newly revealed mechanism of PLD1 for HIF-1α stability. We suggest that PLD1 does not act as a molecular platform by losing affinity to the proteins under hypoxia. This argument is based on the observation that (1) PLD1 did not associate with either PHD, HIF-1α or VHL in hypoxia, (2) the PH domain of PLD (PLD-PH), which was able to interact with PHD, HIF-1α and VHL and mechanistically acted as intact PLD regardless of oxygen concentration, did not lose the ability to destabilize HIF-1α under hypoxia, and (3) HIF-1v-ODD, which is susceptible to oxygen-dependent degradation, but does not interact with PLD1, was resistant to PLD1-mediated HIF-1α degradation relative to intact HIF-1α. These findings suggest another mechanism underlying hypoxic HIF-1α stabilization. Other than inhibition of PHD leading to prevention of association of HIF-1α with VHL, hypoxia might stabilize HIF-1α by disrupting formation of the multi-protein complex for efficient HIF-1α degradation. We currently have no reasonable explanation of how hypoxia enables PLD1 to lose its ability to interact with proteins and consequently regulate HIF-1α stability. Nonetheless, some of the observations made in this study provide information regarding the regulatory mechanism underlying the effects of hypoxia. Given that PLD1-PH was not responsive to hypoxia, other regions in PLD1 may play a role as a negative regulatory domain to prevent interaction with the proteins that are responsive to hypoxia. In addition, this hypoxia effect has nothing to do with the hydroxylation status of HIF-1α since hypoxia dissociated a HIF-1α mutant resistant to hydroxylation from PLD1. In accordance with these findings, chemical hypoxia did not mimic the hypoxia effect, as indicated by the data demonstrating that PHD inhibitor increased PLD-HIF-1α association, thus ruling out involvement of PHD in the effects of hypoxia. HIF-1α is one of the most compelling therapeutic targets for treatment of tumors growing in hypoxic microenvironments [32-35]. Indeed, PLD1-PH effectively degraded HIF-1α, even under hypoxia. Tumor hypoxia is associated with disease progression, resistance to conventional cancer therapies and poor prognosis. Interestingly, PLD1-PH suppressed tumor progression and expression of HIF-1α and its target genes. Thus, the PH domain of PLD1 may be useful in development of effective anti- HIF-1α peptide therapeutics against cancers.

**Figure 8:**
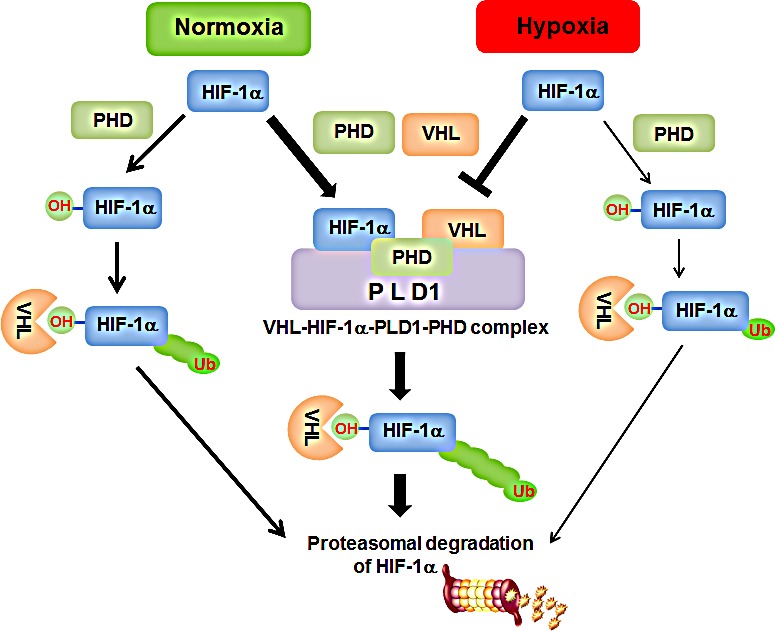
Proposed model of PLD1-mediated HIF1-α stability regulation Undernormoxia, PLD1 directly associates with HIF-1α, PHD2 and VHL, thereby coordinating dynamic assembly of PLD-HIF-1α-PHD-VHL, facilitating PHD/VHL mediated polyubiquitination and consequent proteasomal degradation of HIF-1α. Without PLD1, HIF-1α degradation through PHD/VHL would be retarded. Under hypoxia, interactions of PLD1 with the proteinsare disrupted for unknown reasons and PLD1-independent HIF-1α degradation can occur via PHD/VHL, despite hypoxic suppression of PHD activity. This degradative process might occur with less efficiency thanPLD1-independent HIF-1α degradation in normoxia, in which PLD activity is intact. Thickness of the arrows represents relative contribution to degradation of HIF-1α.

Our findings indicate that PLD1 plays dual roles in the regulation of HIF-1α, while PLD1 activity accelerates the translation of HIF-1α, resulting in up-regulation of the protein, PLD1 protein itself induces efficient degradation of HIF-1α via promotion of the assembly of a HIF-1α-PHD-PLD1-VHL protein complex. These findings suggest a new role in the regulation of HIF-1α in which hydroxylation and ubiquitylation are intimately associated enzymatic activities in a complex. This model allows for further regulation of HIF-1α through restriction of complex association (Figure 8). PLD1 may be a major regulator for determination of the steady state levels of cellular HIF-1α in normoxia. The opposite dual roles of PLD1 may make it possible to exert fine control of HIF-1α protein at various concentrations. Considering that transcription factors often compete with each other with respect to transcription cofactors, the amount of a transcription factor is important to elicit a specific biological effect. In addition, PLD1 might play a role in acceleration of termination of HIF-1α signals as soon as PLD activation signals are turned off, which is an essential mechanism for strict and rapid regulation of biological responses. In conclusion, we revealed a novel role of PLD1 as a crucial oxygen-dependent regulator of HIF-1α stability through regulation of complex formation.

## EXPERIMENTAL PROCEDURES

### Cell culture and transfection

HEK293, MCF-7, MDA-MB-231, MDA-MB-361, HCT116, HT29 cells and PLD1 null MEF were cultured in Dulbecco's modified Eagle's medium (DMEM) with 10% heat-inactivated fetal bovine serum (FBS) and 1% antibiotic-antimycotic (Invitrogen) at 37°C in a 5% CO_2_/95% air incubator. Hypoxia was induced by exposing cells to 1% O_2_/5% CO_2_ balanced with N_2_ using a hypoxic chamber (Forma, Costa Mesa, CA, USA). Cells were transfected using Lipofectamine Plus or Lipofectamine 2000 (Invitrogen) according to the manufacturer's instructions.

### Pulse-chase assay

HEK293 cells were incubated with methionine/cysteine-free DMEM (Invitrogen) containing 2% FBS for 1 h. Cells were pulsed with 300 μCi of [^35^S]methionine-cysteine per well in methionine/cysteine-free DMEM (Invitrogen) in the presence of MG132 (2.5 μM) for 4 h. The cells were then washed in PBS and chased by replacing DMEM with 10% FBS containing 100 μg/mL _L_-methionine and 500 μg/mL _L_-cysteine. The cell lysates were subsequently incubated with HIF-1α antibody in the presence of the protein A sepharose beads. Radiolabeled HIF-1α protein was assessed by SDS-PAGE and autoradiography.

### GST pull-down assay

GST-vector and GST-fusion proteins were expressed using *Escherichia coli* BL21 cells. GST-fusion proteins were applied to glutathione-sepharose (GSH) 4B beads (Amersham). In vitro-translated protein was generated using the TNT quick coupled transcription/translation systems (Promega). GSH bead-conjugated GST-fusion proteins were subsequently mixed with cell lysates or in vitro-translated proteins at 4°C for 1 h, after which they were washed five times with PBS containing 1% triton-X 100. Finally, proteins were eluted in SDS sample buffer and analyzed by SDS-PAGE.

### Immunoblot and immunoprecipitation

Cells were lysed in passive-lysis buffer (Promega) followed by gentle sonication. Cell lysates were incubated with a suitable antibody in the presence of protein A sepharose beads (Amersham). In the hydroxylated-HIF-1α peptide competition assay, biotinylated wild-type or proline hydroxylated-peptides (corresponding to HIF-1α residues 556–574) were synthesized (American Peptide Company) and then dissolved in sterile water (500 μg/ml). The peptide was subsequently added to the immunoprecipitation mixture, after which the following antibodies were used for immunoblot and immunoprecipitation: HIF-1α, α-tubulin, GFP, HA, myc epitope, PHD2, Elongin C and GST (Santa Cruz Biotechnology); hydroxylated-HIF-1α (Cell Signaling Technologies); FLAG (Sigma); VHL (BD Bioscience). A polyclonal anti-PLD antibody that recognizes both PLD1 and PLD2 was generated as previously described [36].

### Luciferase assay

Cells were seeded in 24-well plates and then transiently transfected with pGL2-HRE (HIF-1α-responsive element)–Luc and pRL-TK (internal control). The activities of Firefly and Renilla luciferase in the cellular extracts were subsequently measured using a Dual-Luciferase Assay kit (Promega, WI) according to the manufacturer's instructions.

### Xenograft study

A mouse xenograft model was established using 6-week old BALB/c nude mice (Central Lab Animal Inc., Seoul, Korea). The HT29 cells expressing vector or PLD1-PH were suspended in serum-free medium and then injected subcutaneously into the mouse. After xenografts started growing, the volume and weight of the tumors was measured. The animal protocol used in this study was reviewed by the Pusan National University–Institutional Animal Care and Use Committee (PNU-IACUC) for ethical procedures and scientific care and approved (approval number PNU-2009-0024).

### Luciferase assay

Cells were seeded in 24-well plates and then transiently transfected with pGL2-HRE (HIF-1α-responsive element)–Luc and pRL-TK (internal control). The activities of Firefly and Renilla luciferase in the cellular extracts were subsequently measured using a Dual-Luciferase Assay kit (Promega, WI) according to the manufacturer's instructions.

### Statistical Analyses

The results are expressed as the mean ± S.D. of the number of determinations indicated. Significant differences among means were identified by ANOVA. A *P* value <0.05 or 0.01 was considered to indicate statistical significance.

## SUPPLEMENTARY MATERIAL FIGURES


