# Supplementary material for: Phospholipase D1 protein coordinates dynamic assembly of HIF-1α-PHD-VHL to regulate HIF-1α stability
Source: Oncotarget. 2014 Oct 21;5(23):11857–72. doi: 10.18632/oncotarget.2613 (PMC4323006; doi:10.18632/oncotarget.2613)
Supplement: Supplementary file 1 [file oncotarget-05-11857-s001.pdf]

Phospholipase D1 protein coordinates dynamic assembly of HIF-1α-PHD-VHL to regulate HIF-1α stability

Supplementary Material

Supplemental Experimental Procedures

Materials

Desferrioxamine (DFX) was purchased from Sigma. Cycloheximide (CHX) and MG132 were purchased from AG scientific. 5-fluoro-2-indolyl des-chlorohalopemide (FIPI) was purchased from sigma. The siRNA for hPLD1 (Genbank accession number: U38545; nucleotides 1486–1506, AAGGUGGGACGACAAUGAGCA) and hPLD2 (Genbank accession number: AF033850; nucleotides 2665 to 2685, AAUGGGGAUGCGGAUAAAGGC) were purchased from Dharmacon Research Inc. Mammalian expression plasmids encoding GST-HIF-1α, the truncated GST-HIF-1α contructs, A various GFP-tagged PH domains were gift from Dr. Sung Ho Ryu (Pohang University of Science and Technology). GFP-HIF-1α and flag-HIF2 were kindly supplied by Dr. Kyu-Won Kim (Seoul National University). HA-Elongin C was obtained from Dr. Pierre G. Lutz (INSERM).

PLD activity assay

PLD activity was assessed by measuring the formation of [^{3}H]phosphatidylbutanol, the product of PLD-mediated transphosphatidylation, in the presence of 1-butanol. Cells were seeded in six-well plates at 2×10^5/well, and then incubated in the presence of 3 µCi/ml [^{3}H] myristic acid. After overnight labeling, the cells were washed and then 0.3% 1-butanol was added. The extraction and characterization of lipids by TLC were done as previously described [1].

RNA isolation and real-time quantitative PCR (qPCR)

Total RNA was isolated by the TRIzol reagent (Invitrogen). First-strand cDNA was synthesized using AMV-RTase (Promega), and real-time q-PCR was performed by using

SYBR green PCR kit (QIAGEN). The q-PCR forward and reverse primer sequences, respectively are followed: for HIF-1α 5'- GAACGTCGAAAAGAAAAGTCTCG-3' and 5'-CCTTATCAAGATGCGAACTCACA-3'; for GAPDH, 5'-GTGGTCTCCTCTAGCTTCAAC-3' and 5'-TCTCTTCCTCTTGTGCTCTTG-3.

Immunohistochemistry

Tumor tissues were fixed in 10% buffered formalin (Sigma). The specimens were embedded in paraffin and 5 μm thick-cutted sections were routinely stained with haematoxylin and eosin (HE). The sections were stained with antibodies to HIF-1α and VEGF. And then, the antigen antibody complex was visualized by the addition of peroxidase-conjugated universal secondary antibody.

Viability assay

The stable cells were seeded and incubated at 37°C for the indicated time. Afterwards, the cells were incubated for 3 h with phenol red-free medium containing 500 μg/ml of 3-(4,5-dimethylthiazol-2-yl)-2,5-diphenyl tetrazolium bromide (MTT, Sigma). The MTT-containing medium was removed after 3 h of incubation. The incorporated dye was dissolved in 100 μl/well of DMSO, after which the plates were read at a wavelength of 540 nm using an ELISA reader. Absorbance in the treated cells was expressed as percentage of the control.

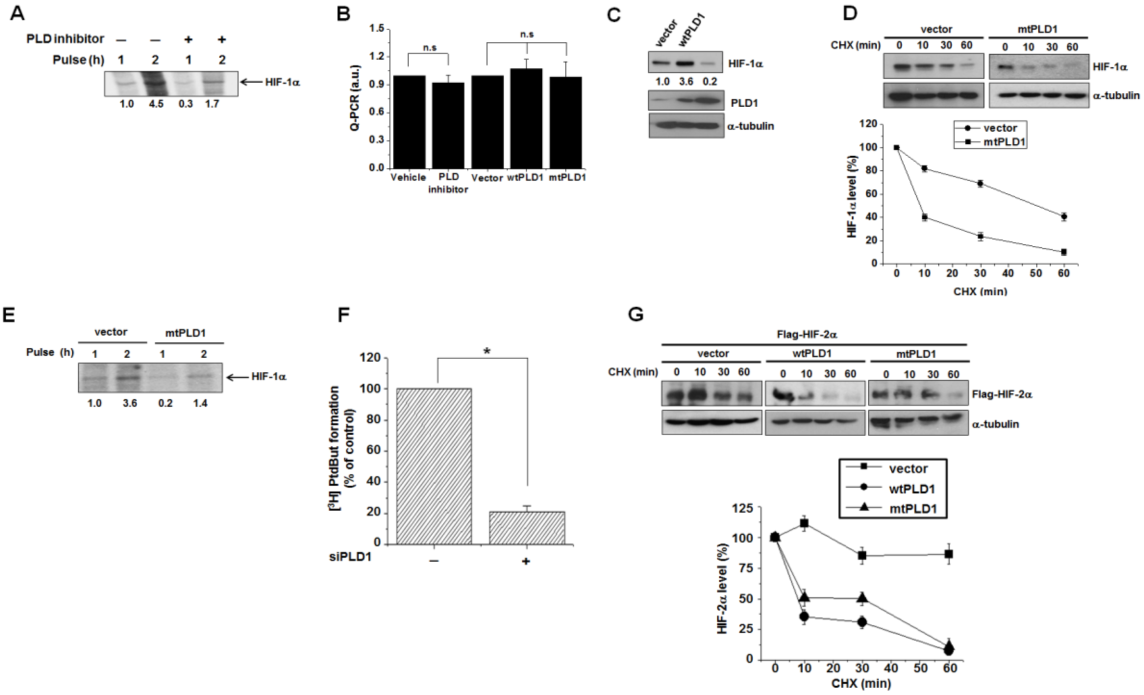

Figure S1: Related to Figure 1. PLD1 plays dual roles in regulation of HIF-1α.

(A) Pulse assay of HIF-1α. HEK293 cells were pulse labeled with [^{35}S]methionine-cysteine for the indicated times in the presence of MG132 (2.5 μM) and PLD inhibitor (FIPI, 100 nM). Lysates were immunoprecipitated with antibody to HIF-1α and assessed by autoradiography. Relative band intensity was quantified. Data are representative of three independent experiments.

(B) q-PCR assay of HIF-1α from HEK293 cells treated with the PLD inhibitor or transfected with wtPLD1 or mtPLD1. n.s, nonsignificant, Data are presented as the mean ± SD of four independent experiments.

(C) Immunoblot (IB) analysis of HIF-1α from HEK293 cells transfected with wtPLD1 or mtPLD1. The band intensity was quantified. The levels of HIF-1α.to α-tubulin were normalized. Data are representative of three independent experiments.

(D) HEK293 cells were transfected with mtPLD1 and incubated under hypoxia for 4 h and then reoxygenated and in parallel treated with CHX for 30 min. The lysates were analyzed by immunoblotting, after which the band intensity was quantified. The levels of HIF-1α.to α-tubulin were normalized. Data are representative of three independent experiments.

(E) HEK293 cells were transfected with the indicated constructs and pulse labeled with [^{35}S]methionin-cystein for the indicated time in the presence of MG132 (2.5 μM). The lysates were immunoprecipitated with antibody to HIF-1α and assessed by autoradiography. Data are representative of three independent experiments.

(F) PLD activity assay in HEK293 cells transfected with PLD1 siRNA. *, $P < 0.05$ versus control siRNA. Data are presented as the mean ± SD of four independent experiments.

(G) IB analysis of HIF-2α in HEK293 cells cotransfected with flag-HIF-2α and wt or mt PLD1 in the presence of CHX. The lysates were analyzed by immunoblotting, after which the band intensity was quantified. The levels of HIF-2α.to α-tubulin were normalized. Data are representative of three independent experiments.

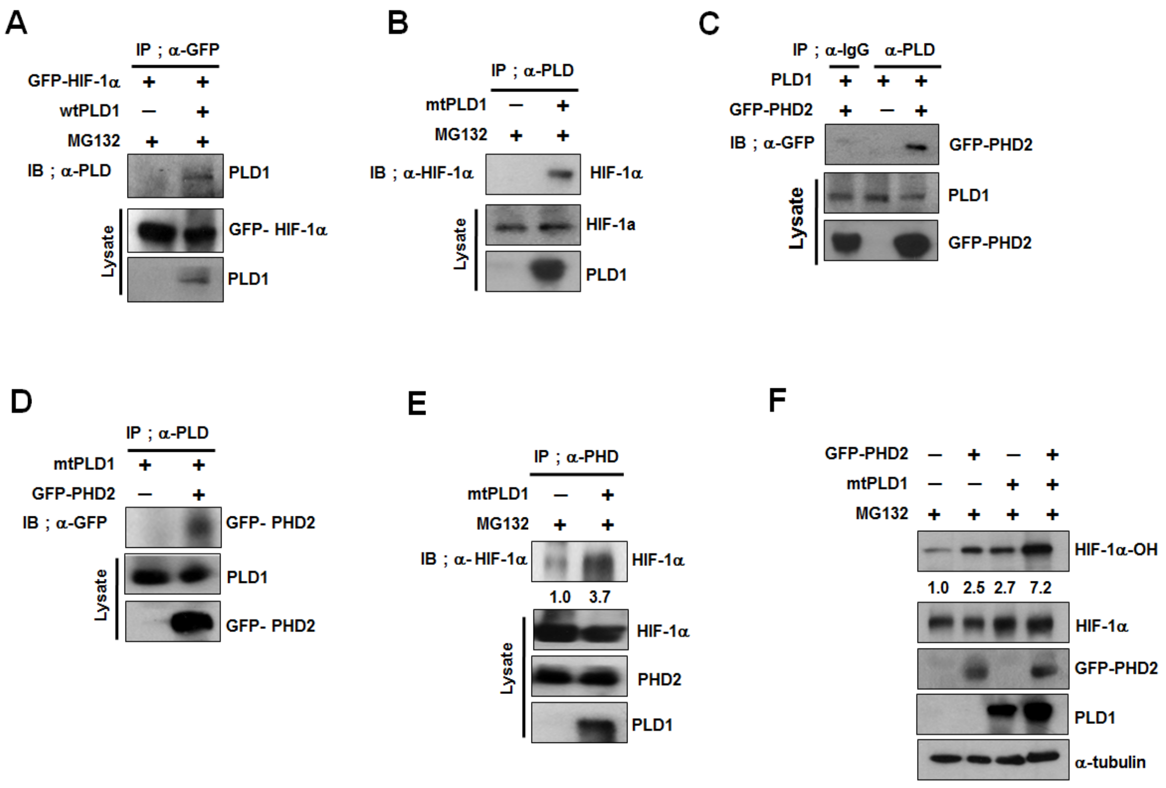

Figure S2: Related to Figure 2. mtPLD1 interacts with HIF-1α and PHD2, and promotes prolyl hydroxylation of HIF-1α.

(A**)** IP assay for the interaction of wtPLD1 with exogenous HIF-1α in the presence of MG132. Data are representative of three independent experiments.

(B) IP assay for the interaction of mtPLD1 with endogenous HIF-1α in the presence of MG132. Data are representative of three independent experiments.

(C) IP assay for the interaction of PLD with exogenous PHD2. Data are representative of three independent experiments.

(D) IP assay for the interaction of mtPLD1 with exogenous PHD2. Data are representative of three independent experiments.

(E) Effect of mtPLD1 on the interaction of PHD2 with HIF-1α in the presence of MG132. The lysates were analyzed by immunoblotting, after which the band intensity was quantified. Data are representative of three independent experiments.

(F) IB analysis of hyroxylated-HIF-1α in HEK293 cells cotransfected with PHD2 and/or mtPLD1 in the presence of MG132. The lysates were analyzed by immunoblotting, after which the band intensity was quantified. Data are representative of three independent experiments.

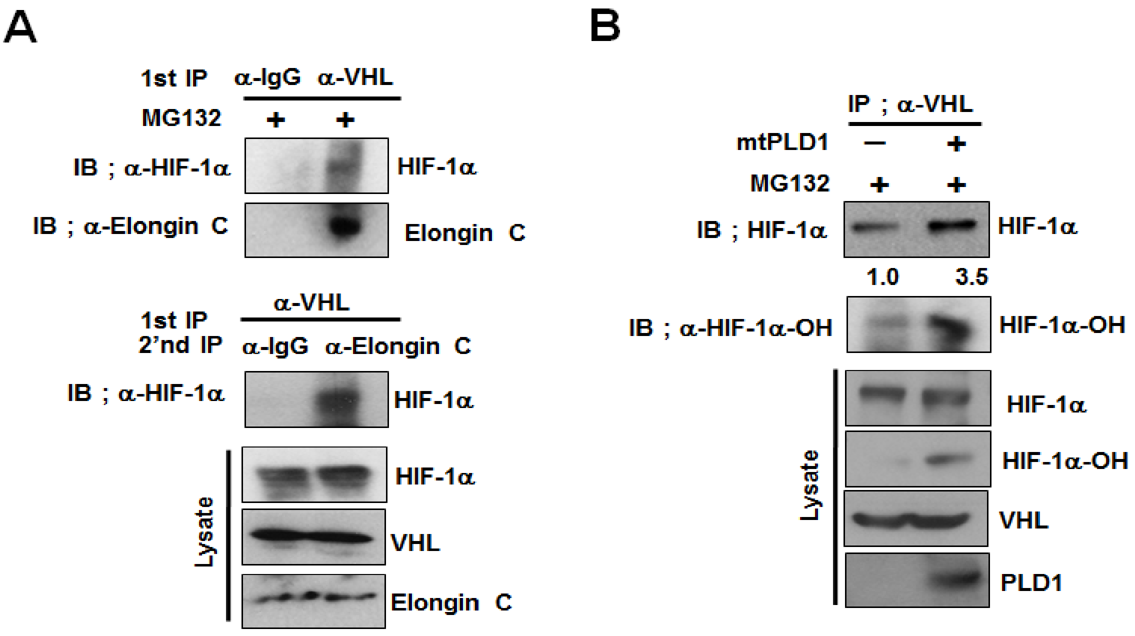

Figure S3: Related to Figure 3. HIF-1αis present in VHL and Elongin C immune complex.

(A) Coimunoprecipitation (CoIP) assay of lysates from HEK293 cells in the presence of MG132. The lysates were immunoprecipitated with anti-VHL antibody and immunoblotted with the indicated antibody. The proteins released from primary immunoprecipitates were reimmunoprecipitated with antibody to Elongin C and analyzed by immunoblot with anti-HIF-1α. Data are representative of three independent experiments.

(B) Effect of mtPLD1 on the interaction of VHL with HIF-1α or hydroxylated HIF-1α in the presence of MG132. The lysates were analyzed by immunoblotting, after which the band intensity was quantified. Data are representative of three independent experiments.

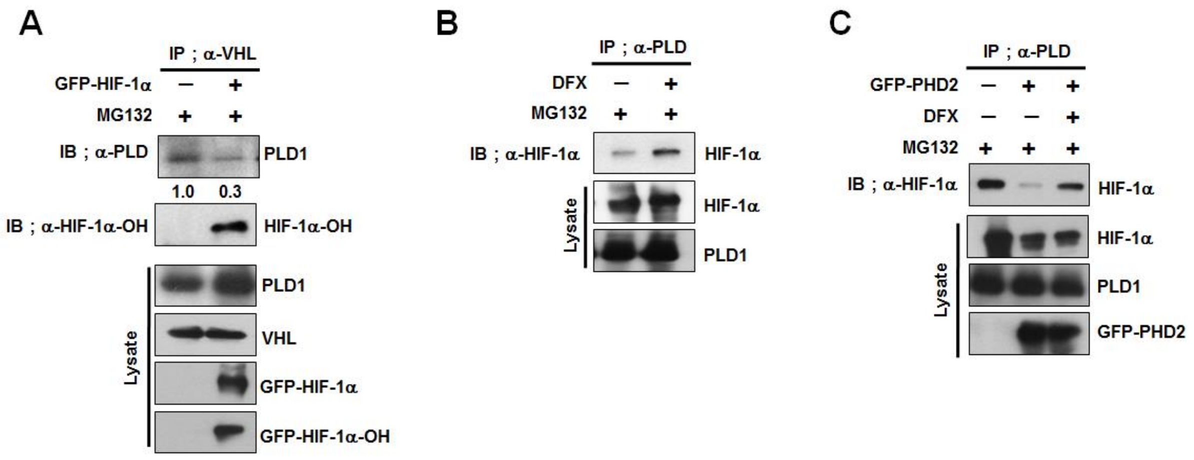

Figure S4: Related to Figure 4. PLD1 enhances interaction of VHL with hydroxylated HIF-1α.

(A) Effect of exogenous HIF-1α on the interaction of VHL with PLD1 in the presence of MG132. The lysates were analyzed by immunoblotting, after which the band intensity was quantified. Data are representative of three independent experiments.

(B) IP assay was performed to examine the effect of DFX (4 h) on the interaction of PLD1 with HIF-1α in the presence of MG132. Data are representative of three independent experiments.

(C) IP assay was performed to examine the effect of DFX on the interaction of PLD1 with HIF-1α in the presence of MG132. Data are representative of three independent experiments.

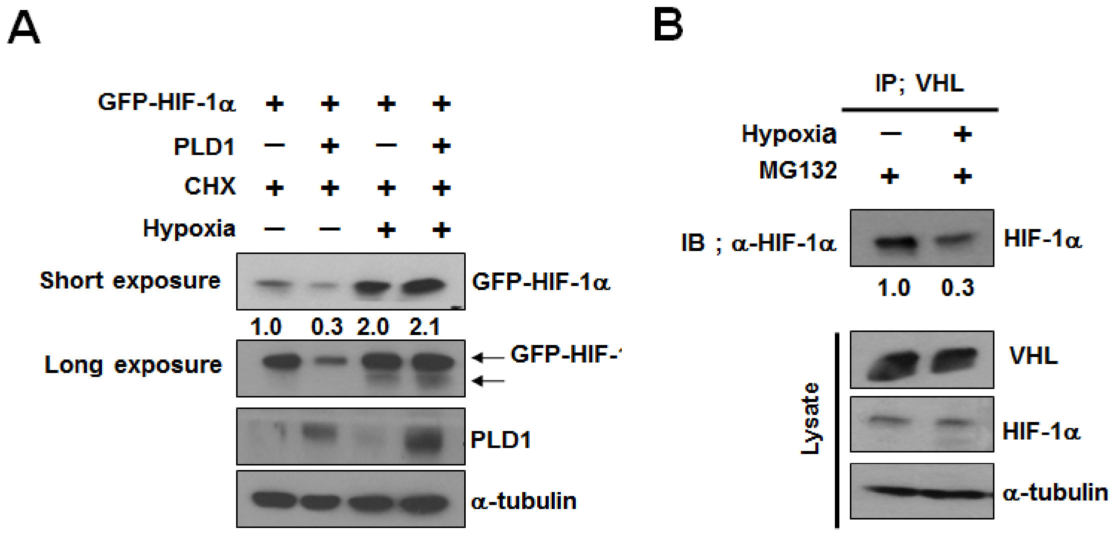

Figure S5: Related to Figure 5. PLD1 does not degrade HIF-1α protein under hypoxic condition.

(A) Effect of PLD1 on the stability of HIF-1α under normoxia and hypoxia in the presence of CHX. The lysates were analyzed by immunoblotting, after which the band intensity was quantified. Data are representative of three independent experiments.

(B) Effect of hypoxia on the interaction of VHL with HIF-1α in the presence of MG132. The lysates were analyzed by immunoblotting, after which the band intensity was quantified. Data are representative of three independent experiments.

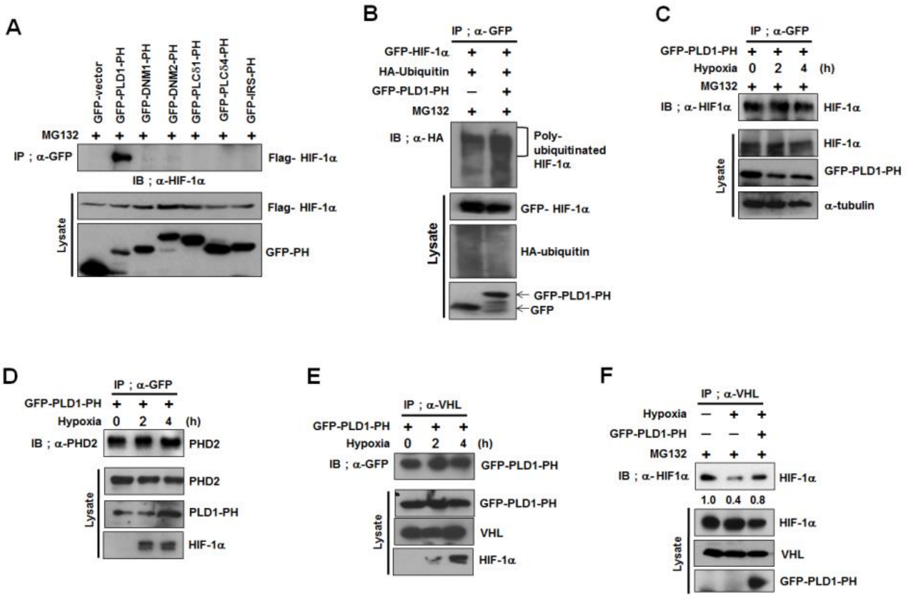

Figure S6: Related to Figure 6. PH-domain of PLD1 associates with HIF-1α, PHD2, and VHL under normoxia and hypoxic condition.

(A) Effect of PH domain of various proteins on the interaction with HIF-1α in the presence of MG132. Data are representative of three independent experiments.

(B) IP assay was performed to measure the ubiquitination of exogenous HIF-1α by PLD1-PH in the presence of MG132. Data are representative of three independent experiments.

(C-E) Effects of hypoxia on PLD-PH-HIF-1α (C), PLD1-PH-PHD2 (D), and VHL-PLD1-PH interactions. Data are representative of three independent experiments.

(F) Effect of PLD1-PH on the interaction of VHL with HIF-1a under hypoxia in the presence of MG132. The lysates were analyzed by immunoblotting, after which the band intensity was quantified. Data are representative of three independent experiments.

(G) Effect of PLD1-PH on the PLD activity. NS, nonsignificant. Data are presented as the mean ± SD of four independent experiments.

(H) IB analysis of lysates from HEK293 cells cotransfected with flag-HIF-2α and/or PLD1-PH in the presence of CHX for the indicated time. The levels of HIF-2α.to α-tubulin were normalized. Data are representative of three independent experiments.

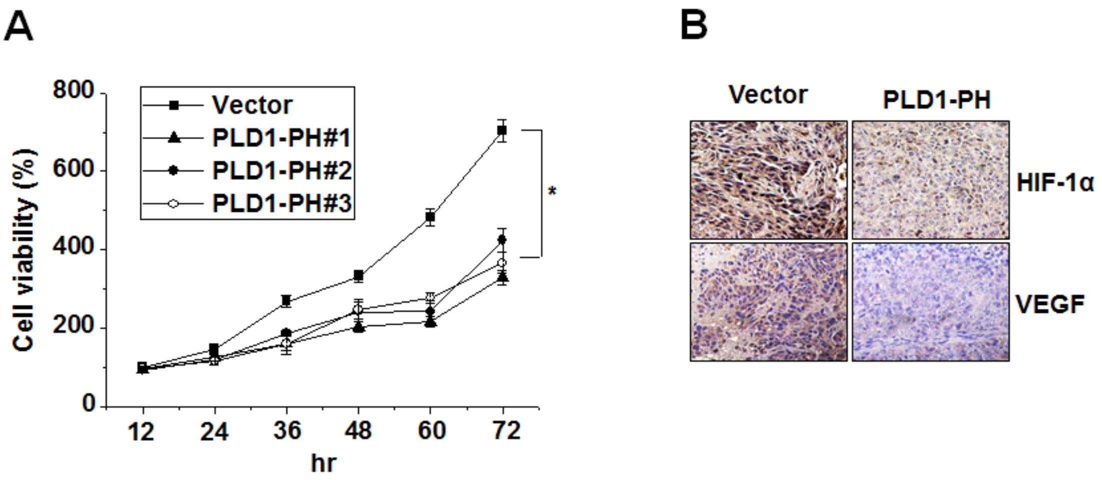

Figure S7: Related to Figure 7. Effect of PLD1-PH on the cell viability and the expression of HIF-1α and its target proteins. (A) The cell viability was measured in three clones of stable HT29 cells expressing PLD1-PH for the indicated time, as analyzed by MTT assay. *$p<0.05$ versus vector cells, Data are presented as the mean ± SD of four independent experiments. (B) Immunohistochemistry in tumor tissues derived from the xenografted mice injected with vector or PLD1-PH stable cells. Data are representative of three independent experiments.

SUPPLEMENTAL REFERENCES

1. Kang DW HW, Park MH, Ko GH, Ha WS, Kim KS, Lee YC, Choi KY, Min DS. Rebamipide abolishes Helicobacter pylori CagA-induced phospholipase D1 expression via inhibition of NFκB and suppresses invasion of gastric cancer cells. Oncogene. 2013; 35(30):3531-3542.
